# Problems with visual statistical learning in developmental dyslexia

**DOI:** 10.1038/s41598-017-00554-5

**Published:** 2017-04-04

**Authors:** Heida Maria Sigurdardottir, Hilda Bjork Danielsdottir, Margret Gudmundsdottir, Kristjan Helgi Hjartarson, Elin Astros Thorarinsdottir, Árni Kristjánsson

**Affiliations:** Department of Psychology, University of Iceland, Reykjavík, Iceland

## Abstract

Previous research shows that dyslexic readers are impaired in their recognition of faces and other complex objects, and show hypoactivation in ventral visual stream regions that support word and object recognition. Responses of these brain regions are shaped by visual statistical learning. If such learning is compromised, people should be less sensitive to statistically likely feature combinations in words and other objects, and impaired visual word and object recognition should be expected. We therefore tested whether people with dyslexia showed diminished capability for visual statistical learning. Matched dyslexic and typical readers participated in tests of visual statistical learning of pairs of novel shapes that frequently appeared together. Dyslexic readers on average recognized fewer pairs than typical readers, indicating some problems with visual statistical learning. These group differences were not accounted for by differences in intelligence, ability to remember individual shapes, or spatial attention paid to the stimuli, but other attentional problems could play a mediating role. Deficiencies in visual statistical learning may in some cases prevent appropriate experience-driven shaping of neuronal responses in the ventral visual stream, hampering visual word and object recognition.

## Introduction

Developmental dyslexia is one of the most common learning disabilities, with a prevalence rate of up to 17.5%^1^. An estimated 14% of Icelandic students have problems with reading and 4% are severely impaired^2^. Dyslexia is generally considered to have linguistic roots, and there is strong consensus among researchers that dyslexic readers are impaired in phonological processing^3–10^. The relative importance of phonological factors in reading nonetheless depends on the orthographic depth of a language^11^, and people can exhibit satisfactory performance on phonological tests even if they cannot read fluently^12, 13^. Factors other than phonological processing might therefore also contribute to dyslexia.

In recent years the role of visual perception and visual attention in reading difficulties has increasingly been highlighted. Researchers have looked for possible defects in low-level vision supported by primary visual cortex or subcortical structures as well as high-level vision supported by cortical regions of the dorsal or ventral visual streams.

A large body of work on dyslexia has explored the role of visual deficits arising from the workings of the magnocellular-dorsal (MD) visual pathway (for a review, see e.g. refs 14–16; for a critical approach, see e.g. refs 17–24). This can refer to visual deficits arising from anywhere between the retina and the posterior parietal cortex^14^ as the dorsal visual stream, culminating in the posterior parietal cortex, depends largely (but not exclusively) on magnocellular contributions^25^. For example, abnormalities in the magnocellular layers of the lateral geniculate nuclei have been reported in dyslexic readers^26^, problems with visual motion perception (thought to primarily rely on the MD pathway) are found in dyslexic readers and such problems predict future reading development^27, 28^ (see also ref. 29), and motion direction-discrimination training aimed at specifically enhancing the sensitivity and timing of MD pathway processing shows promise in remediating reading difficulties^30^.

There is also mounting evidence that people with dyslexia have problems with visual attention, primarily supported by a fronto-parietal network^31–33^ sometimes included in the MD pathway^34^. This includes a deficit in the automatic orienting of attention (including sluggish attentional shifting) and problems with sustained focused attention^35, 36^ (see also e.g. refs 37–39). Problems with selective visual spatial attention are found in future poor readers before reading acquisition, suggesting a causal link between attention and reading problems^40^, and improving the attentional skills of dyslexic children appears to improve their reading abilities^41^ (see also ref. 34). It has been proposed that fronto-parietal attentional networks sequentially prioritize locations corresponding to successive letters, and that even mild impairments of such attentional mechanisms could give rise to reading problems^42^ (for more reviews on visual and visuo-attentional factors in dyslexia, see e.g. refs 13, 43–46; for a critical approach, see ref. 47).

Unlike the magnocellular/dorsal stream, the ventral stream (sometimes referred to as the parvocellular-ventral or PV stream) has often been assumed to be intact in dyslexic readers (see e.g. ref. 48). However, some of the tasks on which this conclusion is based, such as the detection of high-pass filtered rings^48^, detection of fixed sinewave gratings^49^, or the sensitivity to static visual form coherence^50^, might mainly tap into the workings of the lower portion of the ventral visual stream. We have previously demonstrated that people with dyslexia are impaired not only at recognizing visually presented words but also faces and other visually complex objects^51^. While we emphasize that this does not automatically argue against other theories of the causes of dyslexia, the possibility remains that reading problems in dyslexia might in some ways be a manifestation of a more general high-level visual deficit. This, again, is consistent with the fact that particular regions in the ventral visual stream – thought to play a crucial role in the visual recognition of words and other complex objects – have consistently been shown to be hypoactive in both children and adults with dyslexia^52^ (see ref. 51 for an extended discussion) and in prereading children with a familial risk for dyslexia^53^. The difficulties that dyslexic readers have with visual recognition presumably stem from these functional abnormalities, but what causes the functional abnormalities is unclear.

One possibility is that the visual experience of dyslexic readers is inefficient in shaping their visual system. Neurons high up in the ventral visual stream are tuned to increasingly complex combinations of visual features, and visual experience is thought to play a fundamental role in establishing neural representations that are selective for feature combinations supporting object recognition^54^ (for a review on the effects of experience on visual object processing, see ref. 55). This is seen in word-selective ventral stream regions such as the visual word form area (VWFA; see e.g. refs 56–58 for reviews). The region’s responses to nonsense letter strings (e.g. cvgzm, wchse) depend on how often the letter combinations occur in the person’s native language^59^, and experience with statistical regularities of novel letter-like shapes leads to increased activation of this region^60^ (see also refs 57, 61, 62). Visual experience appears to build neurons tuned to words or word fragments longer than individual letters^59, 62–64^ which could support rapid parallel processing of multiple letters in both words and pseudowords of which typical readers are capable, but dyslexic readers are not^65, 66^.

Learning to respond selectively to an object might require us to pick up the statistical regularities of the visual world and could in essence amount to learning which image features tend to co-occur^54^. Visual statistical learning is the ability to extract regularities from the visual environment and can occur without intent or awareness^67, 68^. While visual statistical learning is often considered an instance of a domain-general mechanism, statistical learning is subject to modality- and stimulus-specific constraints^69–71^.

Visual statistical learning may build an efficient object recognition system with neural mechanisms that extract useful feature combinations based on simpler visual features that tend to co-occur (e.g. letter combinations or word fragments). Impaired visual statistical learning would leave neurons less selective for such feature combinations, affecting the recognition of complex visual stimuli, including written words. If people with dyslexia are impaired at picking up which visual features tend to go together, neurons in the ventral visual stream normally shaped by such learning will not effectively support visual word and object recognition. The primary hypothesis tested here is therefore that people with dyslexia have poor capacity for visual statistical learning.

Research on the sensitivity of dyslexic readers to statistical regularities in text has yielded mixed results. Their sensitivity has been reported to be greater^72^, poorer^73, 74^, and similar^9^ to that of typical readers. There is some evidence for impairments of implicit or automatic learning in dyslexia (for comparison of implicit learning and statistical learning, see ref. 75), although, again, the results are mixed; Lum, Ullman, & Conti-Ramsden^76^ performed a meta-analysis of procedural learning as measured by serial reaction time (SRT) tasks and found that while there were reports of both impaired and intact learning, people with dyslexia have, on average, worse procedural learning abilities than controls. Neural correlates of this reduction or absence of learning in a serial reaction time task have also been reported for dyslexic readers^77^. Recently, Gabay, Thiessen, & Holt^78^ found evidence for impaired auditory statistical learning (of syllables and tones) in developmental dyslexia. Spencer, Kaschak, Jones, & Lonigan^79^ have also found that statistical learning (as assessed by an auditory word segmentation task and a visual sequence-learning task) accounts for a unique proportion of variance in literacy-related skills (oral language, vocabulary knowledge, and phonological processing) in a sample of typically developing children. Visual statistical learning has also been shown to predict the acquisition of literacy in Hebrew as a second language^80^.

Arciuli and Simpson^81^ found a positive link between visual statistical learning and reading ability in the general population. Their task involved visual statistical learning of relationships between complex visual objects (cartoon aliens), but as our previous research shows that reading problems are associated with poor recognition of such complex visual objects^51^, their finding may reflect suboptimal visual processing of the individual items themselves, but recognition of individual items was not tested. The rapid serial visual presentation used is also of some concern given reports that reading problems are associated with a processing deficit for rapidly presented stimuli^82^.

Our primary question is whether people with dyslexia have problems with visual statistical learning. We directly compare the performance of individuals with dyslexia and individuals with no reading difficulties on a well-established visual statistical learning test modeled on previously published tasks (e.g. refs 60, 83). We predict that dyslexic readers will be less likely than typical readers to learn visual statistical regularities. We test temporal visual statistical learning of novel visual shapes that are not visually complex and should be easily recognizable individually. These stimuli were specifically chosen because previous functional neuroimaging results have shown that the temporal visual statistical learning of such shapes affects object-selective and word-selective regions of the ventral visual stream^60^, regions that in turn overlap with hypoactive regions in people with dyslexia^52^. We present the shapes at a slow rate to minimize interference from potential problems with rapid temporal processing.

Attention plays an important role in visual statistical learning^84, 85^. Visual statistical learning of unconnected shapes only occurs at an attended location^84^. Even when all shapes are shown at the same locaction, visual statistical learning only occurs for selectively attended shapes, not for unattended (but nevertheless seen) shapes^85^; however, as long as the shapes are attended, the learning of their statistical relationships can occur automatically, without intent or awareness^85^. High-level regions of the ventral visual stream, functionally abnormal in dyslexic readers (see discussion in ref. 51), are functionally and structurally connected to regions of the dorsal visual stream involved in spatial and feature-based attention^86, 87^ – the same regions that appear to modulate or even gate the access of visual information to ventral stream regions^31, 86^. As neurons in high-level ventral stream regions mainly process attended visual stimuli^31^, inattention could prevent appropriate experience-dependent neural plasticity in these regions. Given the high comorbidity of dyslexia and attention deficit hyperactivity disorder^88, 89^ (ADHD) and visual attentional problems associated with dyslexia (see discussion above), we additionally test whether potential problems with visual statistical learning in dyslexia are mediated by disrupted attentional function.

## Results

### Reading Abilities

#### ARHQ-Ice

Dyslexic readers reported a greater history of reading problems than typical readers, as indicated by their significantly higher scores on the ARHQ-Ice screening test (independent samples t-test, *t*(*63*) = 13.851, *p* < 0.001, *d* = 3.228; Table 1). All dyslexic participants had higher scores on the ARHQ-Ice than their matched typical readers.

**Table 1:** Descriptive statistics for dyslexic and typical readers.

	Dyslexic Readers		Typical Readers	
	Mean	(S.D.)	Mean	(S.D.)
**Nonverbal Intelligence**				
Block Design	51.24	(10.843)	51.22	(11.235)
Matrix Reasoning	18.84	(4.226)	20.41	(3.578)
**Behavioral Assessment**				
* Childhood ADHD symptoms	25.22	(13.177)	12.05	(8.860)
* Current ADHD symptoms	16.19	(8.993)	10.19	(5.296)
**Reading Abilities**				
* ARHQ-Ice	0.62	(0.123)	0.28	(0.084)
* IS-FORM Common Words/Minute	76.24	(14.717)	110.32	(24.976)
* IS-FORM Common Word Accuracy (%)	91.30	(7.305)	97.05	(3.110)
* IS-FORM Uncommon Words/Minute	48.86	(11.579)	78.27	(17.073)
* IS-FORM Uncommon Word Accuracy (%)	76.58	(13.167)	92.65	(4.926)
* IS-PSEUDO Pseudowords/Minute	34.76	(9.656)	55.19	(12.499)
* IS-PSEUDO Pseudoword Accuracy (%)	60.68	(17.894)	83.26	(8.990)
**Visual Tests**				
* Familiarization Phase: Jiggle d’	4.43	(0.698)	4.88	(0.633)
Familiarization Phase: Jiggle c	0.52	(0.242)	0.45	(0.212)
* Shape Recognition (%)	95.89	(6.298)	98.65	(1.785)
* Visual Statistical Learning (%)	67.61	(19.295)	78.23	(18.002)

Significant differences (independent samples t-tests) between dyslexic and typical readers are marked with an asterisk (*).

#### Reading measures

Dyslexic readers tended to perform more poorly on the IS-FORM and IS-PSEUDO reading tests than typical readers (Table 1). Dyslexic participants read considerably fewer common word forms (independent samples t-test, *t*(*58*) = 7.151, *p* < 0.001, *d* = 1.663), uncommon word forms (independent samples t-test, *t*(*63*) = 8.671, *p* < 0.001, *d* = 2.016), and pseudoword forms (independent samples t-test, *t*(*72*) = 7.869, *p* < 0.001, *d* = 1.829) per minute than typical readers. In addition, dyslexic readers had a lower proportion of accurately read word/pseudoword forms on all word lists than typical readers (independent samples t-tests; common word forms: *t*(*49*) = 4.401, *p* < 0.001, *d* = 1.024; uncommon word forms: *t*(*46*) = 6.952, *p* < 0.001, *d* = 1.617; pseudoword forms: *t*(*53*) = 6.856, *p* < 0.001, *d* = 1.595). Real word forms and pseudoword forms distinguished equally well between dyslexic and typical readers (see similar effect sizes).

### Nonverbal Intelligence

The performance of dyslexic and typical readers did not significantly differ on tests of non-verbal intelligence (Table 1), neither for Block Design (independent samples t-test, *t*(*72*) = 0.011, *p* = 0.992, *d* = 0.002) nor Matrix Reasoning (independent samples t-test, *t*(*72*) = 1.722, *p* = 0.089, *d* = 0.401).

### ADHD

Eleven dyslexic participants (29.7%; four reported currently taking medication for treating the condition) but no typical readers reported a previous ADHD diagnosis. Other studies have reported that 18–42% of children with reading disorders also meet diagnostic criteria for ADHD^88^ so our sample of dyslexic readers is representative in this regard. Compared to typical readers, dyslexic participants had significantly higher scores on the ADHD screening test for both current (independent samples t-test, *t*(*58*) = 3.497, *p* = 0.001, *d* = 0.813) and childhood (independent samples t-test, *t*(*63*) = 5.030, *p* < 0.001, *d* = 1.173) symptoms of ADHD (Table 1).

### Familiarization Phase: Jiggle Detection

A signal detection analysis on jiggle detection during familiarization revealed that the discriminability index *d*’ was higher for typical than dyslexic readers (independent samples t-test, *t*(*72*) = 2.945, *p* = 0.004, *d* = 0.624; Table 1). Dyslexic readers may not have been as attentive to the familiarization stream as typical readers, or this might reflect impairments in visual motion perception^90–92^. There was no significant difference in the response criterion *c* of the two groups (independent samples t-test, *t*(*72*) = 1.257, *p* = 0.213, *d* = 0.292; Table 1).

It should nonetheless be emphasized that mean discriminability indices for both groups were very high, indicating good performance of both dyslexic and typical readers on the jiggle task. As suggested by an anonymous reviewer, given the effects that corrections on tail behavior can have on estimating *d*’, particularly with higher *d*’ (cf. ref. 93), the group differences were further explored with a Bayesian model (see ref. 94). The results of this analysis were in accordance with our previous analysis, i.e. the 95% credible intervals (highest posterior density intervals) for *d*’ of the two groups did not overlap, while the 95% credible intervals for *c* were highly overlapping.

### Shape Recognition

Both groups correctly identified the base shapes on a high percentage of trials (dyslexic readers: M = 95.9%, typical readers: M = 98.6%; chance level: 50.0%). This group difference was nevertheless significant (independent samples t-test, *t*(*42*) = 2.564, *p* = 0.014, *d* = 0.596). People with dyslexia may be somewhat disadvantaged at recognizing novel shapes, in addition to impairments in recognizing faces and other complex within-category objects^51^.

### Visual Statistical Learning

**Figure 1:**
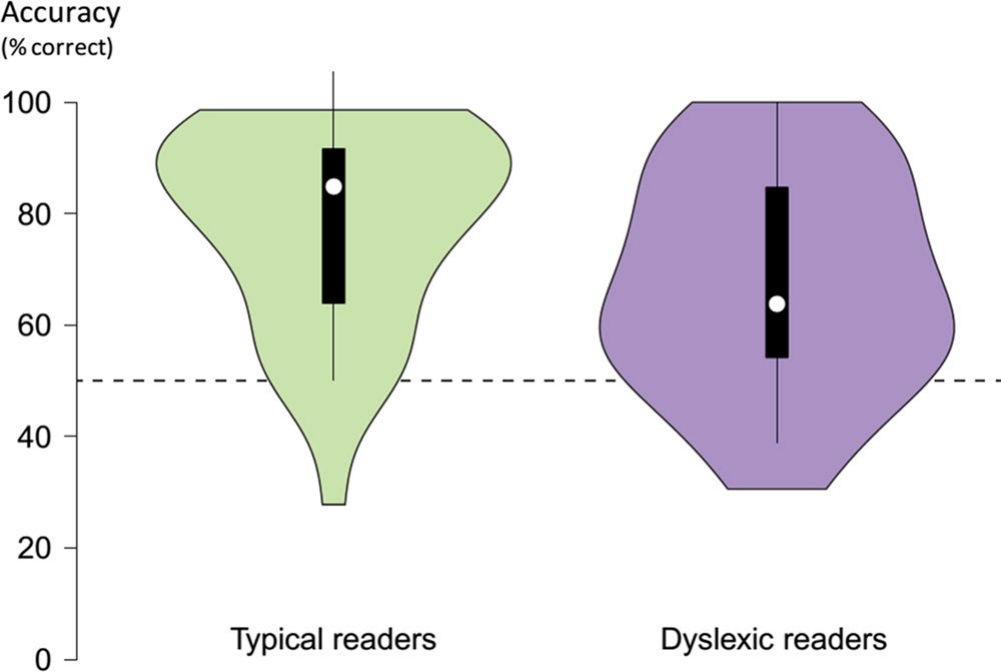
Performance of typical (green) and dyslexic readers (purple) on the visual statistical learning test. Expected chance performance is 50% (dashed line). White discs show the medians. Box limits indicate the 25th and 75th percentiles. Whiskers extend 1.5 times the interquartile range from the 25th and 75th percentiles. Polygons represent density estimates of data and extend to extreme values. This violin plot was created with BoxPlotR^122^.

Dyslexic readers correctly identified significantly fewer base pairs during the visual statistical learning test than typical readers (Table 1; Fig. 1; independent samples t-test, *t*(*72*) = 2.449, *p* = 0.017, *d* = 0.569), suggesting that dyslexic readers on average had lower capacity for learning statistical regularities in the familiarization stream than typical readers. There was also a significant positive correlation between accuracy on the visual statistical learning test and reading speed across the entire sample (*r*(72) = 0.255, *p* = 0.029; the positive correlation with reading accuracy did not reach significance, *r*(72) = 0.189, *p* = 0.106). This correlation with reading speed was lower and non-significant when either subgroup was considered alone (dyslexic readers: *r*(35) = 0.008, *p* = 0.963; typical readers: *r*(35) = 0.148, *p* = 0.383). The correlation was therefore mainly carried by group membership, i.e. whether or not participants had been diagnosed with dyslexia.

#### What drives the difference in visual statistical learning?

While there were no significant differences between the two groups on either measure of non-verbal intelligence, group differences in Matrix Reasoning would have been significant with a less stringent one-tailed test. To see whether group differences in visual statistical learning might be mediated by differences in intelligence, we therefore performed a two-stage hierarchical regression with performance on the visual statistical learning test as the dependent measure. Hierarchical regression provides a framework of model comparison where regression models including and excluding particular variables of potential interest are compared. As neither measure of nonverbal intelligence takes obvious precedence over the other, scores on Matrix Reasoning and Block Design were both entered at stage one of the regression. Group membership (dyslexic or typical reader) was entered at stage two. At stage one, non-verbal intelligence did not significantly contribute to the regression model (F(2,71) = 1.915, p = 0.155, *R* ^*2*^ = 0.051). Adding group membership at the second stage significantly improved the model (model change: F(1,70) = 6.243, *p* = 0.015, *R* ^*2*^ = 0.078), indicating that a dyslexia diagnosis explained variance in visual statistical learning not explained by nonverbal intelligence.

Another possibility is that dyslexic participants are worse than typical readers at recognizing individual shapes and that this, in turn, leaves them less able to recognize their pairing. We therefore again performed a hierarchical regression with performance on the visual statistical learning test as the dependent measure, now with single shape recognition entered at stage one and group membership at stage two. At stage one, single shape recognition did not significantly contribute to the model (F(1,72) = 2.883, p = 0.094, *R* ^*2*^ = 0.038). Introducing group membership at the second stage significantly improved the model (model change: F(1,71) = 4.147, *p* = 0.045, *R* ^*2*^ = 0.053). Differences in single shape recognition abilities of dyslexic and typical readers therefore could not account for the observed differences in visual statistical learning.

A third possibility is that typical readers paid more attention to the shapes during familiarization, possibly affecting visual statistical learning. Previous work has shown that the input to visual statistical learning is gated by attention^84, 85^. Jiggle detection performance provides a measure of attention and motion perception during familiarization. We performed a hierarchical regression with *d*’ and response criterion *c* entered at stage one (one was not assumed to take precedence over the other) and group membership at stage two. Jiggle detection performance did not significantly contribute to the regression model at stage one (F(2,71) = 0.230, p = 0.795, *R* ^*2*^ = 0.006). Adding group membership during the second stage significantly improved the model (model change: F(1,70) = 5.351, *p* = 0.024, *R* ^*2*^ = 0.071). Attention (or motion perception) as measured by jiggle detection did not account for statistical learning differences.

Jiggle detection may not capture all attentional problems of the participants. Dyslexia and attention deficit hyperactivity disorder (ADHD) partly share a genetic etiology, are frequently comorbid, and such comorbidity is associated with more severe deficits and outcomes^88^. We performed a final four-stage hierarchical regression to assess the possibility that problems with visual statistical learning experienced by dyslexic readers are mediated by their attentional problems as assessed by ADHD diagnosis and symptoms. A formal diagnosis of ADHD was entered at the first stage of the regression. Current ADHD symptoms were assumed to be a better measure of attentional state at the time of testing and were therefore entered at the second stage, and childhood symptoms of ADHD at the third stage. Group membership was entered at the fourth stage. At stage one, ADHD diagnosis contributed only marginally significantly to the regression model (F(1,72) = 3.919, *p* = 0.052, *R* ^*2*^ = 0.052). At stage two, adding current symptoms of ADHD did not significantly improve the model (model change: F(1,71) = 0.430, *p* = 0.514, *R* ^*2*^ = 0.006). At stage three, adding childhood symptoms of ADHD explained an additional 10% of the variation in visual statistical learning and significantly improved the model (model change: F(1,70) = 8.100, *p* = 0.006, *R* ^*2*^ = 0.098); at this stage, childhood symptoms were the only significant independent contributor to the model. Adding group membership at stage four did not lead to further significant improvements of the model (model change: F(1,69) = 0.722, *p* = 0.398, *R* ^*2*^ = 0.009). Deficits in visual statistical learning experienced by dyslexic readers might therefore be mediated by their attentional problems.

#### Implicitness of learning

24 dyslexic and 21 typical readers reported no awareness, 5 dyslexic and 6 typical readers reported vague awareness, and 8 dyslexic and 10 typical readers reported some awareness of the statistical pattern in the visual statistical learning familiarization phase. Participants gave their answers after they had been informed of the existence of shape pairs, so true awareness is likely overestimated. The distribution of answers across the three categories of awareness did not significantly differ between dyslexic and typical readers (Pearson’s chi-squared test, χ^2^(1, *N* = 74) = 0.513, *p* = 0.774).

For typical readers, there was no indication that performance on the visual statistical learning test phase rose with the level of reported awareness (accuracy for participants reporting: no awareness: 80.2%; vague awareness: 74.3%; some awareness: 76.5%; between-subjects ANOVA: F(2, 34) = 0.296, *p* = 0.746, ${\eta }_{p}^{2}$ = 0.017). This is in agreement with previous studies suggesting that visual statistical learning can occur without explicit awareness of the underlying statistical structure^85^. For dyslexic readers, there was a non-significant trend towards a rise in performance on the visual statistical learning test phase with the level of reported awareness (accuracy for participants reporting: no awareness: 63.2%; vague awareness: 76.1%; some awareness: 75.5%; between-subjects ANOVA: F(2, 34) = 1.873, *p* = 0.169, ${\eta }_{p}^{2}$ = 0.099).

It is worth noting that the group difference in visual statistical learning was very large for participants who reported no awareness of statistical structure (*d* = 0.926), but was virtually non-existent for those who reported either vague or some awareness (*d* = 0.003). We looked at this more formally by running an additional two-step hierarchical regression with visual statistical learning as a dependent variable. We entered dyslexia (no: 0; yes: 1) and awareness (no: 0; yes: 1) at stage one of the model. At this stage, the model as a whole was significant (F(2,71) = 3.334, p = 0.041, R^2^ = 0.086); dyslexia was a significant independent contributor to the model (p = 0.021) while awareness was not (p = 0.407). We entered a dyslexia x awareness interaction term at the second stage of the model. Adding this interaction term only marginally significantly improved the model (model change: F(1,70) = 3.762, p = 0.056, R^2^ = 0.047). A marginally significant interaction and self-reports of unknown validity should give the reader cause for interpreting these results with some caution. They are nonetheless in line with the possibility that people with dyslexia have a problem with implicit but not explicit learning of visual statistics.

## Discussion

People with dyslexia on average showed poorer visual statistical learning of simple nonsense letter-like objects than matched typical readers. Our study was partly inspired by previous work on the effects of visual experience on the visual word and object recognition system of the ventral visual stream, and we interpret the results within this general framework.

The response properties of neurons within high-level ventral stream regions are normally shaped by unsupervised (non-feedback driven) learning of which visual features tend to go together^60, 95–99^. Visual statistical learning of stimuli as those used here has previously been shown to shape the response properties of word-selective ventral stream regions sensitive to word-form statistics, regions that are hypoactive in people with dyslexia^52, 57, 59–62^. We propose that problems with visual statistical learning are one (but certainly not necessarily the only) plausible cause for these functional abnormalities.

This suggestion needs to be further established in future studies, especially in light of the moderate effect size (see Fig. 1). There is considerable overlap between the performance of dyslexic and typical readers, some of which may be due to measurement uncertainty but some of which could be due to genuine individual variability; it is neither necessarily so that all dyslexic readers are bad at visual statistical learning nor does it have to be the case that all typical readers are particularly good at visual statistical learning. However, at least some of the overlap between the two groups could be explained by two different learning mechanisms contributing to performance: visual statistical learning, which can occur without intent or awareness^67, 68^, as well as a more explicit type of learning. Restricting the analysis to the participants whose performance was presumably driven purely by the former learning mechanism, i.e. the majority of people who reported no explicit awareness of any pattern or rule in the order of the sequence of shapes, indeed revealed a large group difference. In future studies it might be advisable to attempt to directly manipulate awareness, e.g. by informing some but not all participants of the existence of a statistical structure prior to learning.

How specific are the observed problems with visual statistical learning? Previous research as well as the current study has shown that temporal visual statistical learning of novel shapes is not well explained by general cognitive ability such as intelligence or working memory, with the exception of a modest correlation (r = 0.23) with Raven’s Advanced Progressive Matrices^71^. Note that the relationship between some measures of general cognitive abilities, such as processing speed (which is reduced in dyslexic readers^100^) is unknown. The observed problems also do not reflect a deficit in reinforcement learning as no feedback of any kind was given. They are unlikely to be driven by a deficit in explicit rule learning in the sense that “various mnemonics, heuristics, and strategies are engaged to induce a representational system”^101^ as participants were not informed of any statistical regularities in the familiarization stream, had no incentive for discovering such statistical regularities, were preoccupied with a cover task, and group differences were strongest for those people who reported no awareness of statistical regularities. The deficits could possibly be related to procedural learning where repeated exposure to a task results in improved performance on that task, regardless of whether this exposure is consciously remembered or not^102^. However, procedural learning tasks, such as serial reaction time (SRT) tasks, tend to have an important motor component, and the neural substrates of procedural memory – including the basal ganglia, cerebellum, and other motor-related areas – reflect this^76^. Temporal visual statistical learning of novel nonsense shapes, as in the current study, crucially relies on the medial temporal lobe^103, 104^ which has reciprocal connections with high level regions of the ventral visual stream^105, 106^. In contrast, amnesic patients with bilateral diencephalic or hippocampal lesions exhibited learning of an embedded repeating sequence on a serial reaction time task similar to that of controls^107^, and visual statistical learning appears not to be correlated with learning of a serial reaction time task^71^. Other implicit learning tasks such as artificial grammar learning can also be intact in people with damage to the medial temporal lobe^108^. Visual statistical learning is therefore probably distinct from these other types of learning.

The problems that some people with dyslexia appear to have with visual statistical learning could potentially generalize to other modalities. Gabay, Thiessen, & Holt^78^ found evidence for impaired auditory statistical learning of dyslexic readers, but as there is individual variability in statistical learning even within a group of dyslexic readers, it is unknown whether dyslexic readers who have problems with visual statistical learning also are the ones who have problems with auditory statistical learning. General problems with statistical learning could lead to all sorts of deficits that could then influence reading, e.g. poor learning of statistical mappings between sounds and letters (grapheme/phoneme correspondence regularities) as well as poor learning of visual representations of words and word fragments. However, Siegelman & Frost^71^ found that performance on a visual statistical learning task similar to the one used here was neither correlated with auditory verbal nor auditory non-verbal statistical learning. The domain specificity or generality of statistical learning is an active topic of research and debate (see e.g. ref. 70).

Previous work shows that attention is important for visual statistical learning^84, 85^, and our results are consistent with this although childhood ADHD symtoms appear to be a better predictor of such problems than jiggle detection, previous ADHD diagnosis, or current ADHD symptoms, all of which might be expected to better capture current attentional capabilities.

Jiggle detection should be better on average if spatial attention is maintained at screen center. However, since all four motion directions were relevant and equally likely, feature-based selection of a particular motion direction would have been unhelpful. Jiggle detection may therefore measure space-based attention better than feature- or object-based attention. Turk-Browne, Jungé, & Scholl^85^ demonstrated that the input to visual statistical learning is gated by attention by making people selectively pay attention to a particular feature dimension (red or green) instead of a particular location. When attention is paid to objects, processing of other features belonging to that object should be enhanced^109–111^. For visual statistical learning, it might not be enough to “pay attention” in a broad sense – specifically processing the to-be-learned features (here: shape or form) might be required. In future studies, it might be beneficial to run other tasks, preferably standardized, that measure various aspects of visual attention more specifically.

With regard to ADHD, some dyslexic readers may have managed to behaviorally compensate for an underlying deficit – a deficit best captured by their childhood behavior – which still appears in subtle ways as seen here. It is also possible that childhood symptoms of ADHD really capture the severity of the participants’ history of reading problems, as behavior indicative of ADHD can also be directly indicative of problems with reading (e.g. “did not pay close attention to details or made careless mistakes in work or schoolwork”^112^) or be a consequence of serious reading problems in school (e.g. “left my seat in situations in which it was inappropriate”^112^). If this interpretation is correct, then our suggestion that attentional problems mediate problems with visual statistical learning in dyslexia is less plausible, as it would simply mean that people with a history of more severe reading problems are also the ones that are more likely to have problems with visual statistical learning. This needs to be addressed in further studies. Whether people who have a history of symptoms of ADHD without a history of reading problems would have problems with a comparable visual statistical learning task also needs testing.

In sum, we suggest that dyslexia in some cases involves an impaired ability to automatically pick up visual statistical regularities in the environment. This impairment may be mediated by attentional problems. This should make neurons in the ventral visual pathway less selective for complex visual features, including visual word fragments corresponding to statistically likely letter combinations. This would leave dyslexic readers less able to develop reading strategies that involve rapid parallel processing of multiple letters in words and pseudowords, and might force people to resort to an inefficient letter-by-letter reading strategy as is indeed seen to a greater extent for people with dyslexia than for typical readers^65, 66^.

## Methods

All methods were carried out in accordance with Icelandic guidelines and regulations for scientific studies on human subjects. All experimental protocols were approved by the Icelandic Science Review Board (Vísindasiðanefnd, http://vsn.is) and reported to the Icelandic Data Protection Authority (Persónuvernd, http://www.personuvernd.is). All participants gave informed consent.

### Participants

Participants were 40 adults with a prior diagnosis of dyslexia and 40 adult typical readers. Of the 80 participants, six were immediately resampled; three because button presses were not recorded, two because they dozed off during testing, and one because the person did not report an uncorrected hyperopia until after testing. Three additional participant pairs were excluded (see Methods: Data Analysis section). This left 37 people with dyslexia and 37 typical readers (21 females in each group).

Participants in the two groups were matched so that for each dyslexic participant there was a typical reader of the same gender, age (±5 years), and educational background (three levels: finished high school, finished gymnasium, or finished college at the undergraduate level). All were native Icelandic speakers and reported normal hearing and normal or corrected-to-normal vision. None were color-blind. The mean age for dyslexic readers was 26.8 years (range: 19 to 60 years) and 26.3 for typical readers (range: 18 to 56 years). In each group, 12 individuals had completed the first level of schooling, 20 had completed the second level of schooling, and five had completed the third level.

All participants were volunteers but were invited to partake in a lottery; five randomly selected participants were offered a gift card for a local shopping mall (value: 10.000 ISK, approx. 80 USD). In addition, participants from the University of Iceland could receive partial course credit for participation.

### Test Materials and Procedure

The study took place in a quiet and well-lit room. Several measures and tests were administered. Those analyzed here are measures of reading abilities, of non-verbal intelligence, of ADHD, and three visual tasks, as detailed below. If participants preferred, consent forms and questionnaires were read aloud by experimenters.

#### Reading abilities

Three tests were administered to measure reading abilities: ARHQ-Ice, the IS-FORM reading test, and the IS-PSEUDO reading test. The two reading tests were administered at the end of the session after all other tasks had been completed to prevent frustration with poor reading from affecting dyslexic participants’ performance on other tasks.

#### ARHQ-Ice

The Icelandic version of the Adult Reading History Questionnaire^113, 114^ measured participants’ history of reading difficulties indicative of dyslexia. The ARHQ-Ice yields scores from zero to one, with higher scores suggesting a greater likelihood of dyslexia. A score above 0.43 is a suggested cutoff point when screening for dyslexia. The Icelandic adaptation of the ARHQ is a reliable and valid screening instrument for dyslexia^113^.

#### IS-FORM

The IS-FORM reading test^51^ and the newly developed IS-PSEUDO reading test were used to assess participants’ current reading ability. The IS-FORM consists of word lists, one with 128 common Icelandic word forms and another with 128 uncommon word forms. Participants were asked to read the lists aloud as fast as they could, making as few errors as possible. IS-FORM captures a wide variety of reading ability, and dyslexic and typical readers differ greatly on this measure^51^.

#### IS-PSEUDO

The IS-PSEUDO reading test was developed and administered in the same way as the IS-FORM. Prior research suggests that one of the most sensitive measures of dyslexia involves problems in reading phonologically valid pseudowords because this greatly relies on phonological processing abilities^115, 116^. The measure of interest is the number of pseudoword forms read per minute and the percentage of correctly read pseudoword forms. The IS-PSEUDO contains pseudoword forms that nonetheless look like plausible and pronounceable word forms of nouns, adjectives, verbs, or adverbs in Icelandic. The number of pseudoword forms (128) on the IS-PSEUDO was the same as the number of word forms on either IS-FORM list, and the number of syllables (343) was also kept the same as the number of syllables on either IS-FORM list. The order of the pseudoword forms was originally randomized, and then kept fixed for all participants

#### Nonverbal intelligence

The Block Design (possible score range: 0–68) and Matrix Reasoning (possible score range: 0–26) subtests from the Icelandic version of the Wechsler Adult Intelligence Scale – Third Edition (WAIS-III)^117^ were used to measure nonverbal intelligence. These nonverbal subscales do not rely on reading skills^118^.

#### ADHD

Participants were asked if they had previously been diagnosed with ADHD and answered two questionnaires of ADHD symptoms as defined by the DSM-IV^112^. The first questionnaire is a self-report of current symptoms where the frame of reference is the participant’s behavior in the past six months. The second questionnaire is a self-report of childhood symptoms for ages 5 to 12. Participants get a total score from zero to 54 on each list, and higher scores indicate more ADHD-related symptoms. These questionnaires are reliable and valid screening instruments for ADHD^112^.

#### Visual tasks

Three related visual tasks were performed in order: A visual statistical learning familiarization phase, a single shape recognition phase, and a visual statistical learning test phase. The visual tasks were similar to tasks previously used in the visual statistical learning literature^60, 83, 119^.

#### Visual stimuli

**Figure 2:**
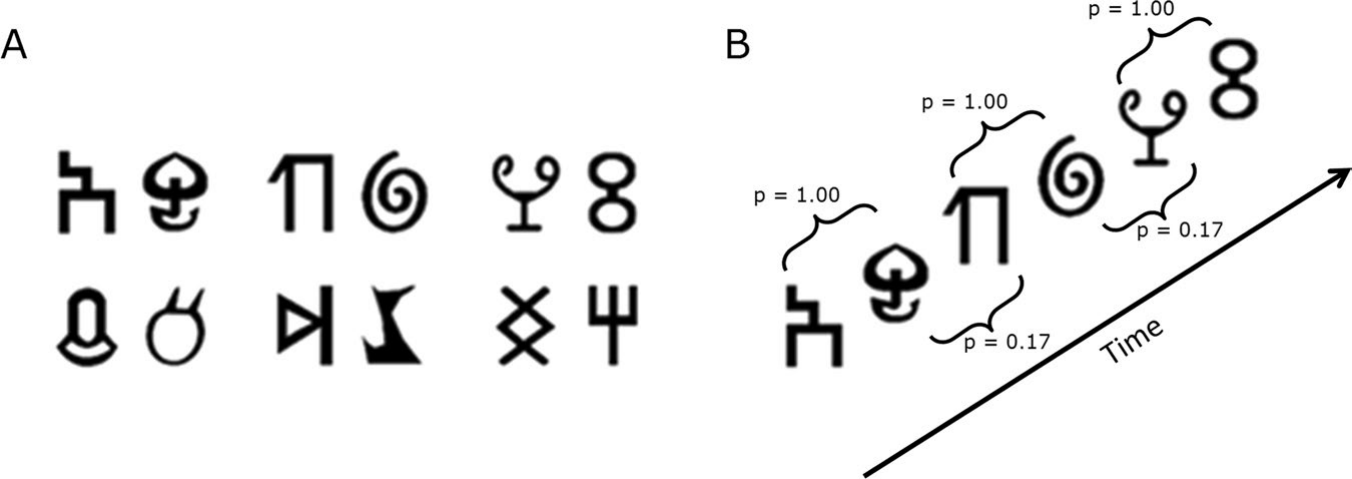
Schematic representation of stimuli and design. (**A**) Sample base pair structure containing randomly selected base shapes. (**B**) During the visual statistical learning familiarization phase, participants were asked to watch a stream of consecutively presented shapes. Transitional probabilities (denoted by *p*) defined base pair boundaries.

The stimuli were 48 novel shapes taken from unfamiliar alphabets. Several different symbols were used to minimize the possibility that results would be driven by idiosyncratic shape characteristics. Fifteen shapes were from the Sabaean alphabet and 13 from the Ndjuka syllabary. The temporal visual statistical learning of Sabaean and Ndjuka glyphs has already been shown to affect regions of the ventral visual stream that correspond to regions hypoactive in dyslexic readers^52, 60^. In addition, 8 shapes came from the Santali alphabet, 7 from the Agathodaimon font and 5 from the Klingon alphabet. Images of the shapes were generated using fonts of the respective alphabets (available at www.omniglot.com and www.fontpalace.com). The maximum height and width of each shape were scaled to be equal using the Image Processing Toolbox for MATLAB (MathWorks). Example shapes are shown in Fig. 2.

For each matched pair of participants, twelve of the 48 available shapes were randomly assigned without replacement to the visual statistical learning familiarization phase (base shapes). These base shapes were also used as stimuli in the subsequent single shape recognition phase and visual statistical learning test phase. For each matched pair of participants, the twelve base shapes were further divided into six base pairs. These six pairs were created by randomly selecting, without replacement, two base shapes out of the twelve original ones until all base shapes had been assigned to a base pair. This created six unique base pairs. Additionally, twelve shapes were randomly picked to be used as foil shapes in the single shape recognition phase.

The shapes used in the visual tasks and the order of their presentation were the same for each matched participant pair (but varied across participant pairs). Each dyslexic participant therefore saw the same shapes in the same order as his or her typical reader counterpart in all visual tasks.

The shapes always appeared in black on a white background and subtended roughly 0.74° in height and 0.55° in width. Participants sat without head restraints approximately 62 cm from the computer monitor. The stimuli were presented on a 17-inch CRT monitor (85 Hz) using PsychoPy^120^.

#### Visual statistical learning familiarization phase

The six base pairs were displayed sequentially in a continuous stream (duration: 1500 ms; ISI: 0 ms; Fig. 2). One base shape was shown at a time in isolation at center. The two shapes of each base pair always appeared consecutively in a particular order during familiarization (Fig. 2). In other words, shape A was always followed by shape B, shape C was always followed by shape D etc. (e.g., ABEFABKLCDEFAB…). Therefore, the initial member of a base pair always predicted the subsequent appearance of the second member of the pair in the familiarization stream. Each base pair was displayed 72 times for a total of 864 trials (pilot tests indicated that such repeated exposure to the base pairs was needed for robust visual statistical learning).

Participants were told that several shapes would appear on the screen, one at a time. They were encouraged to take note of their appearance as they would later on be asked about what the shapes looked like (i.e. in the single shape recognition phase). Participants were not notified on any underlying statistical structure of the familiarization stream and had no knowledge of the visual statistical learning test phase. To keep participants from explicitly focusing on this underlying structure, they performed a cover task during familiarization. Individual shapes would occasionally jiggle: the shapes would move from their center position in a random diagonal direction (top left, bottom left, top right, or bottom right) for approximately 0.2° and then veer back to their original position. The jiggle occurred 650 ms into the trial, lasting 200 ms. From the start of a jiggle, participants had 1500 ms to respond. These jiggle trials occurred randomly in one out of every six shape presentations for a total of 144 trials. Jiggle detection was used both because a similar task had been used in previous research of visual statistical learning of these kinds of shapes^60^ and because pilot studies indicated that the jiggle task allowed robust visual statistical learning of the base pairs. Twelve practice trials at the beginning of familiarization (not analyzed) with three additional shapes that did not appear in any other part of the visual tasks, introduced participants to the cover task.

#### Single-shape recognition phase

The 12 base shapes in the visual statistical learning familiarization phase were pitted against 12 foil shapes that were not presented during familiarization. On each trial, one base shape and one foil shape were displayed one at a time in isolation in the center of the display. The first shape was followed immediately by the second shape (stimulus duration: 1500 ms; interstimulus interval: 0 ms) which was then followed by an empty screen for 3000 ms. Additional 200 ms separated trials. Participants judged which of the two shapes had appeared in the familiarization phase by pressing one of two buttons. Participants had 4500 ms from the initial appearance of the second shape to press one of two keys to specify which of two shapes had appeared during familiarization. Each base shape and each foil shape was displayed four times for a total of 48 randomized trials.

#### Visual statistical learning test phase

Six unique foil pairs were randomly created using the same 12 shapes displayed during familiarization. No single shape was assigned to a foil pair twice. A shape that served as the first member in a base pair would thus become the first member of a foil pair, and the second shape of a base pair would likewise become the second member of a foil pair. Foil pairs were randomly created for each dyslexic/typical reader matched pair.

During each trial, one base pair was displayed along with one foil pair. The shapes constituting each pair were displayed one at a time in isolation (stimulus duration: 1500 ms; interstimulus interval: 0 ms) with a 1000 ms gap separating the base pair from the foil pair. When both pairs had been presented the screen turned white for 300 ms. A 200 ms inter-trial interval separated trials. Participants indicated by keypress whether the first or second shape pair had appeared more often during the familiarization phase. Participants had 4500 ms from the initial appearance of the last shape to respond. Each base pair was pitted against each foil pair on two separate occasions, once with the base pair and once with the foil pair shown first, for a total of 72 trials. Each base and foil pair was presented 12 times. Therefore, any additional statistical learning that might occur during the test phase would be equal for both pair types. Trial order was randomized for each dyslexic/typical reader participant pair. A preference for base pairs over foil pairs indicates visual statistical learning.

#### Implicitness of learning

After completing the visual statistical learning test phase, participants were asked whether or not they had noticed any pattern or rule in the order of the shapes presented during the visual statistical learning familiarization phase. Responses were coded into three categories: 1. Said that they did not notice any pattern or rule in the order of the sequence (no awareness); 2. Said that they noticed a pattern but either could not describe it or the description did not indicate awareness that shapes came in pairs (vague awareness); 3. Said that they noticed a pattern and that they realized that at least some of the shapes came in pairs (some awareness).

### Data Analysis

Three participants did not follow the instructions for the visual tests. One had 199 false positives for jiggle detection during the visual statistical learning familiarization phase and correspondingly her d-prime measure was over three standard deviations below the mean. A second had no correct answers in the single shape recognition phase, over eight standard deviations below the mean. A third reported after the fact that he had responded contrary to instructions during the visual statistical learning recognition phase. Only 11% of his responses were correct which is highly significantly below chance (one-sample binomial test, p < 0.001). This led to the removal of the corresponding subject pairs. Analyses are based on data from the remaining 37 subject pairs (37 participants with dyslexia and 37 typical readers).

The IBM SPSS Statistics package was used to calculate independent samples t-tests, Pearson’s correlations, hierarchical linear regressions, Pearson chi-squared test, and between-subjects ANOVAs. All statistical tests were two-sided (alpha = 0.05). The more traditional signal detection analysis was based on log-linear corrections for extreme values^121^, and the Bayesian statistical inference of the same data was done with RStan, an R interface to Stan. Degrees of freedom for independent samples t-tests were adjusted in cases where Levene’s test for equality of variances was significant.
